# Impaired Social Cognition in Epilepsy: A Review of What We Have Learnt From Neuroimaging Studies

**DOI:** 10.3389/fneur.2019.00940

**Published:** 2019-09-12

**Authors:** Victoria Lyn Ives-Deliperi, Hennric Jokeit

**Affiliations:** ^1^Department of Health Sciences, Neuroscience Institute, University of Cape Town, Cape Town, South Africa; ^2^Department of Neuropsychology, Swiss Epilepsy Centre, Zurich, Switzerland

**Keywords:** neuroimaging, epilepsy, functional magnetic resonance imaging, social cognition, review

## Abstract

**Background:** Social cognition refers to specific mental processes that subserve social interaction. Impaired social cognition has been increasingly reported in patients with epilepsy and negatively affects overall quality of life (QOL). In this article, we will review neuroimaging studies of social cognition in people with epilepsy.

**Methods:** An electronic search of the literature was conducted and 14 studies qualified for inclusion in the review.

**Results:** Although the studies reviewed revealed a varied pattern of neural activations in response to emotion recognition and theory of mind tasks, consensual findings included altered pattern of signal activation in the social cognition network in patients with mesial temporal lobe epilepsy (MTLE) compared to healthy controls and significantly reduced signal activations and functional connectivity within this network in patients with right mesial temporal lobe pathology.

**Conclusion:** This review contextualizes our current understanding of the pathophysiology of impaired social cognition in epilepsy and makes recommendations for further research.

## Introduction

Healthy social functioning serves to enhance quality of life (QOL) by affording meaningful interactions between people and facilitating cooperative relationships. At a more fundamental level, related skills acquired through social learning ensure our very survival (1).

Social cognition encompasses an array of discrete but interacting mental processes. It is conceptualized as a form of information processing that supports the accurate perception and interpretation of the behaviors, thoughts, and feelings of others and guides appropriate responses. A range of sub-processes are involved in social cognition including theory of mind (ToM), emotion recognition (ER), empathy, prosody perception, and body language interpretation (2).

Deficits in social cognition are apparent across a variety of neurological, neurodegenerative, and psychiatric disorders. Although empirical studies on social cognition in epilepsy are limited, this is a growing and important area of research. Impairments in ER and ToM are frequently reported in people with TLE as well as those with extratemporal lobe epilepsy (extra-TLE), and these deficits compromise QOL (3, 4). Impaired ER is a common feature of mesial temporal lobe epilepsy (MTLE) with an average drop-off of 20% in patient scores on related tasks, compared to healthy peers (5). Meta-analyses have shown that early seizure onset and right temporal lobe seizures are associated with the more significant deficits. Tasks of ER and ToM are the most commonly administered in research studies, and there is a need to explore social skills more comprehensively through investigating other domains including empathy, prosody, and body language interpretation in people with epilepsy, as well as the impact of functional deficits on QOL. Further research is also required to better understand the mechanisms of impaired social cognition in this patient population.

People with epilepsy are pre-disposed to social cognitive deficits for a variety of reasons, including psychosocial, neuropsychological, psychiatric, and pathophysiological. Epilepsy-related stigma, role restrictions, over-protectiveness, and fear of seizures all reduce social engagement and compromise the ability to learn and practice social skills. Cognitive impairments in domains of attention, memory, and language, as well as comorbid affective disorders also negatively affect the functional integrity of social cognition (4). In addition to these contributing factors, the network of brain regions subserving social cognition are the same neural circuits affected in temporal and frontal lobe seizure disorders.

Much has been learned about the neural substrates of social cognition in healthy people and those with neurodegenerative diseases. Since social cognition encompasses a variety of skills to be effective, the neural correlates are generally explored at the level of brain networks. Neuroimaging studies have identified neural networks involved in ER and ToM, which involve frontal, temporal, parietal, and occipital cortex, as well as subcortical mesial temporal regions and periaqueductal gray (6, 7). The ER neural network includes brain regions involved in the perception of the human face and regions recruited more selectively in response to emotion. According to a meta-analysis of 105 functional magnetic resonance imaging (fMRI) studies in healthy volunteers, the former includes fusiform gyrus, the fusiform face area, the occipital face area, and posterior superior temporal gyrus, while the latter additionally engages medial frontal gyrus and inferior frontal gyrus, anterior insula, amygdala, cuneus, and lingual gyrus (6). The fusiform face area is understood to mediate low-level processing, attention, and emotional detection of faces (8, 9), while the cuneus and lingual gyrus are activated when emotions are attributed to self and others and attention is directed to face recognition (10). Medial and inferior frontal gyri have shown to be recruited during the processing of emotive facial expression and social and moral behavior, and the anterior insula subserves emotional awareness and empathy of both self and other-orientated body and feeling states (11–13). The right amygdala is most frequently implicated in fear processing (14, 15).

Comparatively little neuroimaging research has investigated impaired social cognition in people with epilepsy, despite the prevalence of related deficits. Further work in this area will help elucidate the mechanisms of disturbed social cognition in this patient population. The aim of this review to summarize what we have learnt from neuroimaging studies conducted to date and make recommendations for further research.

## Methods

**Figure 1:**
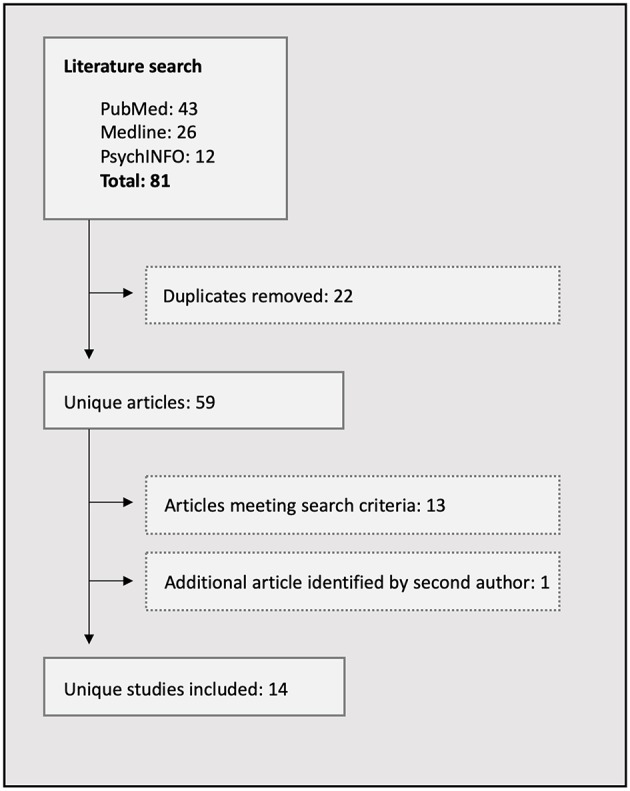
Flowchart of the literature search process.

Literature searches were conducted in PubMed, Medline, and PsychInfo electronic databases by both authors independently. No date restrictions were stipulated, and search terms included the following: (social AND cognition) in the title and (epilepsy) AND (fMRI OR neuroimaging OR MRI) in the abstract. Empirical studies were included in the review if they were published in English and involved neuroimaging of epilepsy patients using ER or ToM tasks. Reference lists of the studies meeting inclusion criteria were searched for additional relevant publications. The search was completed on January 15, 2019, and 14 studies were extracted, all of which are included in the review (Table 1). A flowchart of the search strategy is detailed in Figure 1.

**Table 1:** Neuroimaging studies of social cognition in epilepsy included in the review.

**First author**	**Sample**	**Domain**	**Modality**	**Task**	**BOLD signal increases**
Batut et al. (16)	6 L MTLE 6 R MTLE 15 HC	ER	fMRI	Static faces	Fear vs. neutral HC = **L** IFG, MFG, OL; **R** AMG; **BL** PC LTLE = **L** AMG, CU, UN; **R** IFG, MTL; **BL** PC RTLE = **L** PC, MFG, PHP Sad vs. neutral HC = **L** MTL, PC, SPL, IFG, OL; **R** CU, FFG, ITL, SFL LTLE = **L** MTL, SFL, MFG; **R** FFG, MTL PC, SPL, STG RTLE = **L** MTL; **R** SPL, MTL, SFL Happy vs. neutral HC = **L** CU; **R** PH, MTL, STL LTLE = **L** CU, FFG, IN; **R** MOL RTLE = **L** SFL; **R** MTL, STL
Benuzzi et al. (17)	5 L MTLE 8 R MTLE 14 HC	ER	fMRI	Static facesGender discriminate	Fearful vs. neutral HC = **L** IFL; **BL** OL, LG, TL, FFG LTLE = **R** PH; **BL** IFL, IOL, MOL, TL, FFG RTLE = 0
Benuzzi et al. (18)	2 L MTLE 4 R MTLE	ER	fMRI	Static facesGender discriminate	Fearful faces before vs. after ATL LTLE = **L** OFC; **BL** IPFC, EXST RTLE = **BL** OFC, EXST
Bonelli (19)	26 L MTLE 28 R MTLE 21 HC	ER	fMRI	Static faces	Fearful vs. happy HC = **L** AMG LTLE = 0 RTLE = **BL** AMG
Broicher et al. (20)	12 R HS 16 L HS 18 HC	ToM	fcMRI (ICA)	Dynamic fearful faces	Fearful faces vs. landscapes HC = **L** THL; **R** MTG; **BL** AMG, HP, PG, IFG, IN LTLE = **R** AMG, HP, PG, IFG, MTG, FFG, MTL,PL RTLE = **L** AMG, HP, STG; **R** IFG Group differences in connectivity LTLE = - **L** AMG, HP, STG; **R** IFG RTLE = - **R** AMG, HP, TP, ACC
Ciumas et al. (21)	13 BCECTS 11 HC	ER	fMRI	Static faces	Happy vs. rest BCECTS = **R** LG, CU HC = **R** LG, CU, MOL Fearful vs. rest BCECTS = **R** LG, CU HC = **R** LG, CU, MFG, IFG, STG
Hennion et al. (22)	13 R TLE 13 L TLE 25 HC	ToM	fMRI	Animated shapes	ToM vs. non-ToM group comparisons HC > RTLE = R PC, FFG HC < RTLE = R SFG, DMPFC; LG, CB HC > LTLE = R SFG HC < LTLE = R PHP
Ives-Deliperi et al. (23)	19 R TLE 35 L TLE 6 B TLE 12 exTLE 13 HC	ER	fMRI	Dynamic fearful faces	Fearful faces vs. landscapes HC = **BL** MTL LTLE = **R** MTL RTLE = **L** MTL
Labudda (24)	19 R TLE 18 L TLE 20 HC	ER	fMRI	Dynamic fearful faces	Fearful faces vs. landscapes HC = **BL** MTL, LTL, OL, FL LTLE = **R** MTL, LTL, SFG; **BL** OL RTLE = **BL** PTL, OL Region of interest analysis Lateralized MTL structures in MTLE groups
Schacher (25)	6 R MTLE 6 L MTLE 17 HC	ER	fMRI	Dynamic fearful faces	Fearful faces vs. landscapes HC = **BL** AMG LMTLE = **R** AMG RMTLE = **L** AMG
Steiger et al. (26)	16 R MTLE 17 L MTLE 15 ex TLE 15 HC	ER	fcMRI Seed-based	Dynamic fearful faces	Connectivity analysis within groups HC + EXTLE = **BL** AMG, PAG, IFG, PG, ATL, PTL LMTLE: **R** AMG-PG, aSTG-pSTG RMTLE: 0
Szaflarski et al. (27)	12 L TLE 12 PNES 12 HC	ER	fMRI	Static facesGender discriminate	Group comparisons between HC and TLE Fearful vs. control = PNES + OL, ITL, PL Sad vs. control = PNES – PT Connectivity analysis = LTLE – FL, TL, OL
Toller et al. (28)	18 R MTLE 16 L MTLE 30 HC	ER	fMRI	Dynamic fearful faces	Fearful faces vs. landscapes HC = **BL** AMG, HP; **R** PG, STG, IFG, PT LTLE = **BL** HP; **R** AMG, PT, IFG, PG, MTG, AIN, STG RTLE = **BL** THL; **R** AIN
Vuilleumier et al. (8)	13 HS+AS 13 HS 14 HC	ER	fMRI	Static faces	Fearful vs. neutral HC and HS = **L** ITL, MTL; **R** STG; **BL** IN, AMG, FFG HS+AS = **L** ITL, IFG; **R** AMG, IN

*R, right; L, left; HC, healthy controls; MTLE, mesial temporal lobe epilepsy; PNES, psychogenic non-epileptic seizures; HS, hippocampal sclerosis; AS, amygdala sclerosis; ER, emotion recognition; ToM, theory of mind; fMRI, functional MRI; fcMRI, functional connectivity MRI; AMG, amygdala; HP, hippocampus; IFG, inferior frontal gyrus; MFG, medial frontal gyrus; MTL, mesial temporal lobe; FL, frontal lobe; TL, temporal lobe; OL, occipital lobe; PL, parietal lobe; PG, precentral gyrus; PHP, parahippocampal gyrus; PC, precuneus; CU, cuneus; UN, uncus; FFG, fusiform gyrus; SFL, superior frontal lobe; IPL, inferior parietal lobe; SPL, superior frontal lobe; ITL, inferior temporal lobe; MOL, mesial occipital lobe; STG, superior temporal gyrus; IN, insula; DMPFC, dorsal medial prefrontal cortex; BCECTS, benign childhood epilepsy with central temporal spikes; LG, lingual gyrus; EXST, extra-striatal cortex; THL, thalamus; ACC, anterior cingulate cortex; CB, cerebellum; PAG, periacqueductal gray; EXTLE, extra temporal lobe epilepsy; ATL, anterior temporal lobe; PTL, posterior temporal lobe; PT, putamen; aSTG, anterior superior temporal gyrus; pSTG, posterior superior temporal gyrus; AIN, anterior insula*.

## Results

Functional MRI (fMRI) was conducted in all of the 14 studies included in this review. Two studies used event-related task designs and the remaining 12 used block designs. Two studies included functional connectivity analyses of activated clusters during tasks. Of the 14 studies reviewed, 12 focused on ER/emotional processing (EP), and two explored ToM. Seven studies employed fMRI tasks using static faces expressing emotion and six studies administered a task depicting the dynamic expression of fear. In the two studies investigating the neural mechanisms of impaired ToM, one relied on the dynamic fearful face task and the other used an animated shapes task that has previously been shown to probe explicit and implicit components of the function.

### Neuroimaging Studies Exploring ER Using Static Faces Expressing Emotion

Lesion and neuroimaging studies have demonstrated the critical role of the mesial temporal lobe in recognizing emotion. MTLE affects the hippocampus, the entorhinal cortex, and the amygdala, with discrete amygdala damage observed in 10% of patients (29). Impaired ER is thus common in MTLE, particularly if seizures commence before the age of 5 years. In 2004, Benuzzi et al. explored the effects of unilateral hippocampal sclerosis (HS) on the processing and recognition of the emotion in an fMRI study employing a series of static faces expressing fear (17). BOLD signal activation maps of 8 patients with right MTLE, 5 with left MTLE, and 14 healthy controls (HC) were compared, in response to discriminating gender in fearful and neutral faces. Although no amygdala activations were generated in the comparison of fearful vs. neutral faces, significant unilateral BOLD signal increases were observed in the left inferior frontal gyrus and bilaterally in occipito-temporal regions in HC and in patients with left MTLE. By comparison, there were no significant clusters of activation in patients with right MTLE.

These authors used the same task to investigate possible reorganization of ER network following anterior temporal lobectomy (ALT) in six of these patients (four with right MTLE and two with left MTLE) (18). Improved ER was observed in behavioral testing 6 months after surgery and, in patients with right MTLE, both the occipital and the frontal regions of the right hemisphere were newly recruited in response to fearful faces (18).

In 2006, Batut et al. compared EP of fearful, happy, and sad faces in patients with left and right MTLE compared to HC (16). Results showed differing recruitment of the ER network in patients compared to HC across all emotions. In the *fearful vs. neutral* condition, activations were generated in the left inferior frontal gyrus, mesial frontal gyrus, bilateral occipital lobe, and in the right amygdala and precuneus, in HC. In patients with left MTLE, activations were generated in the same regions but with differing lateralization. Signal increases were noted in the left amygdala, cuneus, and uncus, and in the right inferior frontal and medial gyri and in the precuneus bilaterally. In patients with right MTLE, activations were only noted in the left precuneus, parahippocampal gyrus, and medial frontal gyrus. In the *sad vs. neutral* condition, HC showed activation of the left medial temporal lobe, precuneus, superior parietal lobe, inferior frontal gyrus, and occipital lobe, with right activation of the cuneus, fusiform gyrus, inferior temporal lobe, and superior frontal lobe. Patients with left MTLE showed similar activations with differing lateralization once again, with signal increases in left medial temporal regions, superior and medial frontal lobe, and the right fusiform gyrus, medial temporal lobe, inferior parietal lobe, precuneus, superior parietal lobe, and superior temporal gyrus. Patients with right MTLE showed activations in the left medial temporal lobe and right superior parietal lobe, medial temporal lobe, and superior frontal lobe. Lastly, in the *happy vs. neutral* condition, BOLD signal activations were noted in HC in the left cuneus and right parahippocampal gyrus, medial temporal lobe, and superior temporal gyrus, while left MTLE patients demonstrated activations of the left cuneus, fusiform gyrus and insula, and right medial occipital lobe. Signal increases were noted in patients with right MTLE in the left superior frontal lobe and right medial temporal lobe and superior temporal gyrus.

Static faces expressing fear and happiness were also used as stimuli in a study exploring the utility of fMRI, and amygdala activations in particular, to predict post-operative emotional disturbance in patients undergoing temporal lobectomy (19). BOLD signal changes in response to *fearful vs. happy* faces were compared across three groups; 28 patients with right HS, 26 with left HS, and 21 HC. A block design task was used, displaying a series of pictures, words, and faces (23 fearful faces, 23 happy, and 24 neutral faces) to explore amygdala activation. Significant unilateral BOLD signal increases were observed in the left amygdala in HC, while bilateral amygdala activations were noted in patients with right HS and no significant activations were reported in patients with left HS. Bilateral signal increases in the amygdala of patients with right HS were significantly correlated to post-operative anxiety and depression scores, with right amygdala activation related to increased anxiety and depression after surgery.

Benign childhood epilepsy with centrotemporal spikes (BCECTS) is associated with pathology in the frontotemporal regions and, as such, offers a unique opportunity to study functional pathology in the social cognition network. An event-related potential fMRI study of 13 children with BCETCS was conducted, employing a static face paradigm, to assess the differences in neural activation in response to EP compared to HC (21). The study included two analyses, *happy faces vs. rest* and *fearful faces vs. rest*. Limited activation was generated in the patient cohort in either condition, with significant signal increases limited to the right lingual gyrus and cuneus in response to both. In contrast, widespread signal increases were noted in HC, with additional activations in right medial occipital lobe, medial and inferior frontal lobe, and superior temporal gyrus. Group contrasts revealed reduced bilateral activations in the insula, caudate, and lentiform nuclei in the BCECTS cohort in response to fear processing. The BCECTS cohort also demonstrated increased response time during the task, confirming dysfunction in the social cognitive network. These findings are consistent with reports of altered ER in children with TLE (30).

An earlier study investigated the modulatory influences of the amygdala on distant but connected brain regions during emotion processing of fearful faces, combining imaging, and lesion approaches (8). Activation patterns in various ER conditions were compared between 13 patients with hippocampal and amygdala sclerosis (HS+AS), 13 patients with isolated HS, and 14 HC. HC and HS patients showed a similar pattern of activation in response to *fearful vs. neutral* faces, with BOLD signal increases in the fusiform gyrus, bilaterally in HC and in the left hemisphere in patients with HS, bilateral increase in amygdala and insula, right superior temporal gyrus, and left inferior and medial temporal regions. By contrast, there were no significant signal increases in the HS+AS group, with evidence of only a weak signal change in left inferior posterior temporal gyrus. In a multiple regression analysis across the groups, a significant relationship emerged between the extent of AS and hypoactivation of the visual cortex, left hypothalamus, left hippocampus, bilateral anterior cingulate cortex, and right parietal lobe and superior temporal gyrus, during fear processing.

Szaflarski et al. conducted an fMRI and resting state (RS) connectivity analysis, comparing EP in 12 patients with TLE, 12 patients with psychogenic non-epileptic seizure (PNES), and 24 HC (27). In the fMRI analysis, greater BOLD signal increases were noted in response to happy, neutral, and fearful static faces in the PNES group compared to the TLE cohort. Increased signal was reported in visual, temporal, and parietal regions with decreased activity in response to sad faces in the putamen bilaterally. Seed-based functional connectivity analysis of RS data showed increased functional connectivity in patients with PNES between cerebellar, visual, motor, and frontotemporal regions, as well as between right and left amygdala compared to TLE patients and HC. TLE patients had delayed response times to stimuli in behavioral testing and exhibited hypoactivation of frontal, temporal, visual, and midline brain regions in response to all facial expressions. In addition, no significant correlations were found between the ROIs for the TLE group in RS connectivity analysis.

### Neuroimaging Studies Exploring ER Using Dynamic Fearful Expression

The dynamic presentation of fearful faces has been used in fMRI studies of EP in TLE patients since the development of a related paradigm in 2006 (25). In this blocked design paradigm, patients are presented with fearful expressions of emotion in a series of thriller and horror movie clips, interleaved by control blocks of dynamic landscape video recordings. The perception of motion has been shown to activate amygdala, and together with emotionally laden content, the aim of developing the task was to maximize amygdala reactivity. In the initial study applying the paradigm, significant bilateral BOLD signal increases were generated in the amygdala in 12 HC while 11 of 12 patients with MTLE showed unilateral signal activations in the amygdala, contralateral to the side of seizure onset. Comparable signal asymmetry was noted in hippocampal activations in response to a visual memory task. This study provided not only preliminary evidence of the efficiency of the paradigm to lateralize MTLE but also insights into disturbed functional integrity of the medial temporal lobe during EP in these patients.

These findings have since been replicated (20, 23, 24, 28). In an fMRI study of 37 patients with MTLE (18 left MTLE and 19 right MTLE) and 20 HC, activations were evident in a widespread bilateral network in HC in response to the task. Activated regions included the mesial and lateral temporal lobe, occipital lobe, and frontal lobe (24). Consistent with Schacher's findings, left MTLE patients showed unilateral activations of the right mesial and lateral temporal lobe and superior frontal gyrus, and bilateral activations were noted in posterior regions of the lateral temporal and occipital lobes in patients with right MTLE. A further ROI analysis showed lateralized medial temporal activations in the right hemisphere in patients with left MTLE and in the left hemisphere in patients with right MTLE. Self-reported fear ratings were reduced in the right MTLE cohort.

Lateralized activations of the amygdala were replicated in a more recent study recruiting a larger sample of 60 TLE patients, only 15 of whom had confirmed mesial temporal lobe pathology on MRI. Single-subject analyses were conducted in 35 left TLE patients, 19 with right TLE, 12 with extra-TLE, and 13 HC (23). Right amygdala activations were generated in 23 of the 35 patients with left TLE, and left amygdala activations were reported in 10 of the 19 patients with right TLE. Bilateral amygdala activations were generated in all but one non-epileptic subjects and no clear pattern of signal asymmetry was evident in the extra-TLE group.

The dynamic fearful face task was also applied to investigate whether MTLE is associated with altered empathy-related brain activations in the amygdala, periaqueductal gray, and anterior insula (28). Activation patterns in response to the dynamic task were compared across 16 patients with left MTLE, 12 with right MTLE, 16 with extra-TLE, and 30 HC. Comparable lateralization of amygdala activations was noted in the MTLE group as in previous studies and decreased activations were also noted in periaqueductal gray bilaterally, in the right MLTE group, with preserved right insula activations.

To further interrogate these findings, a seed-based functional connectivity analysis was conducted to assess connectivity between the brain regions activated during the viewing of the fearful face paradigm (26). Widespread bilateral functional connectivity was observed between the amygdala, limbic, cortical, subcortical, and brainstem regions in HC. Specifically, connectivity was evident between the amygdala and periaqueductal gray bilaterally and between right hemisphere inferior frontal gyrus, precentral gyrus, and anterior and posterior temporal lobe. A smaller network of connectivity was noted in patients with left MTLE, involving only the right amygdala and right precentral gyrus, anterior and posterior superior temporal gyrus, and putamen. No significant functional connectivity was present in patients with right MTLE.

### Findings From fMRI Studies Investigating Tom

The dynamic fearful face task was also applied in an fMRI and functional connectivity study investigating which structures within the amygdala network relate to ToM performance (20). The analyses included 16 patients with left HS, 12 with right HS, and 12 HC, and functional connectivity between temporal, frontal, and parietal structures was explored using independent component analysis (ICA). The findings build on evidence of reduced functional and structural connectivity between the hippocampal structures and adjacent brain region in patients with MTLE (31, 32). Once again, bilateral amygdala activation was generated in response to fearful faces in HC, along with bilateral signal increases in hippocampus, precentral gyrus, inferior frontal gyrus and insula, as well as right medial temporal gyrus and left thalamus. Unilateral amygdala activations were observed in patients with left MTLE, contralateral to the lesioned hippocampus, together with right hippocampus, precentral gyrus, inferior frontal gyrus, medial temporal gyrus, fusiform gyrus, medial temporal pole, and palladium. In patients with right MTLE, activations were generated in the left amygdala, hippocampus, superior temporal gyrus, and ipsilateral inferior frontal gyrus. Group differences in connectivity, taking into account duration of epilepsy and IQ, revealed significantly reduced co-activation of the left amygdala, hippocampus, superior temporal gyrus, and right inferior frontal gyrus in patients with left HS compared to HC, and reduced co-activation of the right amygdala, hippocampus, temporal pole, and anterior cingulate cortex in the right HS group compared to HC. Reduced amygdala connectivity with medial temporal pole, right medial temporal gyrus, and left inferior frontal gyrus was also noted in patients with right MTLE compared to those with left MTLE, and this correlated with reduced performance on the Faux Pas test.

The second neuroimaging study to explore the neural underpinnings of impaired ToM in people with epilepsy used an animated shapes fMRI paradigm in 13 patients with right TLE, 13 with left TLE, and 25 HC (22). The task employed both explicit reasoning about mental states and implicit processing of information. Earlier research has shown that patients with MTLE have impaired performances in this task relative to HC, having difficulty in interpreting ToM interactions (20). The task has also been used in neuroimaging studies of ToM in healthy subjects in which the implicit component of the task activated fusiform gyrus, superior temporal gyrus, inferior frontal gyrus, and premotor areas while the explicit component recruited the medial prefrontal cortex (MPFC) and temporal parietal junction (33–35). Different neural activation patterns were generated within the neural networks of the two ToM components in patients with MTLE compared with HC, and these patterns were influenced by the laterality and age at seizure onset. A similar pattern of activation was noted in HC as in earlier studies; however, activations were limited to inferior and medial occipital lobe in patients with right MTLE and no significant activations were noted in patients with left MTLE.

## Discussion

Difficulties in social cognition are common in people with epilepsy, and the earlier the onset of seizures, the more pronounced these deficits (5). The clear overlap between neural networks involved in temporal and frontal lobe epilepsies and the social cognitive network offers a plausible physiological basis for such deficits (6, 7).

The aim of this study was to review what we have learnt from neuroimaging studies of social cognition in people with epilepsy. Since BOLD signal changes generated in fMRI studies are highly specific to the stimuli presented during the in-scanner tasks, meaningful comparisons can only be drawn between studies using the same tasks and comparable protocols. The findings of studies included in this review will therefore be grouped according to (a) those measuring ER/EP to static faces expressing emotion, (b) those measuring ER/EP using dynamic facial expression, (c) those investigating ToM, and (d) those studying connectivity patterns between regions activated in response to fMRI tasks.

The primary findings of the review are as follows: (1) A diverse pattern of BOLD signal increase is reported across studies investigating ER/EP in people with epilepsy and HC using static faces; however, patients with right MTLE generally show hypoactivation of regions in the ER network and performed more poorly on behavioral tasks. (2) More consistent findings are reported across studies investigating ER using a dynamic fearful face task, showing bilateral amygdala activation in HC and lateralized activation in patients with MTLE, contralateral to the side of seizure onset. (3) Studies investigating ToM show reduced signal changes in MTLE patients in the ToM network and reduced connectivity between activated regions, as well as greater recruitment of executive regions in right hemisphere MTLE patients during implicit ToM. (4) Functional connectivity between activated regions during ER is typically reduced in patients with MTLE and particularly so in patients with right MTLE.

### EP Responses to Static Faces Expressing Emotion

The findings of studies reviewed in this paper using static faces expressing emotion report BOLD signal activations in a number of the same regions activated in ER studies of healthy adults (6). The pattern of activations within and across studies, however, varies in terms of lateralization and precise localization. A consistency across all studies was abnormal signal activation within the ER network of patients with right MTLE, and significant correlations between such aberrations and impaired ER on behavioral testing, particularly recognition of fear (16, 17). It has been shown that the greatest impairments in MTLE lie in the recognition of fear and that this impairment is significantly more pronounced for those with right MTLE (3). These findings lend support to the theory that the right MTL is preferentially involved in processing fear and that related lesions disrupt the overall ER network. Right-sided pathology was also shown to relate to greater impairments in young BCECTS patients, who performed more poorly on tasks of emotion recognition and showed reduced activation in the ER network (21). Further to this, isolated amygdala damage was shown to alter activations across the ER network, suggesting that activation of regions involved in EP, in temporal, frontal, and visual cortices, is dependent on the functional and structural integrity of the amygdala (8).

### EP Responses to Dynamic Faces Expressing Emotion

The most outstanding findings across studies using static faces vs. dynamic faces expressing emotion were bilateral signal increases in the amygdala in healthy subjects and unilateral amygdala activation in MTLE patients, in response to the expression of fear. Unlike the studies employing static faces, the dynamic fearful face paradigm generated comparable lateralized amygdala activations across groups in all of the studies reviewed.

Additional activations in response to dynamic fearful vs. scenic video clips in HC included bilateral activation of the hippocampus and right medial temporal gyrus. Activations are also evident in inferior frontal gyrus, superior temporal gyrus, precentral gyrus, and insula in more than one study; however, lateralization of activations in these regions differs. Overall signal activations are reduced in patients with right MTLE.

Right-hemisphere amygdala activations tended to be dominant in functional maps showing bilateral amygdala signal increases in HC and extra-TLE patients, providing evidence for the important role of the right amygdala in vicarious experiences of fear. Stronger activations of the right amygdala in HC and patients with left MTLE correlated significantly with self-reported ratings of fear, and right MTLE patients reported significantly reduced fear ratings (24). An association between right amygdala activation and empathy scores in HC and MTLE patients was also evident, with reduced signal intensity in right amygdala and periaqueductal gray, correlating with reduced empathy scores (28). This has been proposed as a potential mechanism through which right MTLE patients demonstrate reduced responses to fear (28). Together with the overall hypoactivation of other EP regions in patients with right MTLE, the results suggest that the medial temporal lobe may provide fundamental interoceptive input for empathic feelings of fear. Collectively, these findings also suggest that left amygdala is unable to compensate in terms of fear responses in the face of right amygdala damage.

### ToM

Only one study directly investigated the neural underpinning of impairments in ToM in people with epilepsy (22). The same authors previously reported impairments in detecting and understanding faux pas, sarcastic remarks, and mentalistic actions in over 80% of patients with TLE (36). Related impairments have been implicated in abnormal psychosocial functioning and poor QOL in epilepsy (36–38).

Lesion and neuroimaging studies have identified regions of the brain that contribute to cognitive ToM abilities as well as affective ToM abilities. The dorsal MPFC has been shown to be recruited in inferring cognitive mental states and the ventral MPFC in inferring emotional states (39–44). Research findings suggest that both cognitive and affective subcomponents of ToM are impaired in patients with TLE (36). The findings reported by Hennion et al. neuroimaging study confirmed an association between such impairments and task performance and a similar pattern of activation in HC as reported in earlier studies. MTLE patients performed more poorly in the task and showed reduced activation of regions involved in the implicit component of ToM. More intense activations were also evident in regions involved in explicit component of the task in patients with right MTLE (MPFC and temporoparietal junction) (22). The results of this study suggest that the integrity of contralateral mesiotemporal lobe structures plays a more important role in MTLE patients in ToM than remaining spatially connected ipsilateral activity.

### Functional Connectivity Analyses

Resting state connectivity analysis showed reduced connectivity within the ER network in patients with left MTLE compared to patients with PNES and HC (27). This study demonstrated significant connectivity in cerebellar, visual, motor, and frontotemporal regions, as well as between right and left amygdala in PNES patients compared to those with TLE and HC. This finding is consistent with an early investigation measuring functional connectivity between brain regions activated during a social cognition task in HC, which reported significant correlations between signal activations in medial temporal gyrus, temporoparietal junction, anterior insula, lingual gyrus, and cerebellum bilaterally (45).

Results of functional connectivity analysis within the network of activated regions in response to the dynamic task were replicated in two separate studies. Widespread bilateral connectivity was observed between the amygdala, limbic, cortical, subcortical, and brainstem regions in HC in both seed-based analysis (26) and ICA (20). A smaller network of connectivity was noted in patients with left MTLE and no significant functional connectivity was evident in patients with right MTLE. Comparable connectivity patterns in the extra-TLE group to those in HC further suggest that altered patterns of connectivity could not be attributed to seizure activity or AED treatment (26). Similarly, amygdala co-activation with temporal and frontal regions was significantly reduced in patients with right MTLE in ICA (20). Amygdala connections in patients with left MTLE were comparable in strength to those in HC while significantly reduced in right MTLE patients, and these connectivity patterns were strongly associated with scores on a ToM test. These findings emphasize the important role played by medial temporal lobe in ER and ToM in MTLE patients.

## Summary and Conclusion

This review of neuroimaging studies of impaired social cognition in people with epilepsy included studies employing a variety of tasks and imaging paradigms to investigate EP and ToM, reporting variable findings. The majority of studies reviewed reported BOLD signal activation in, and connectivity between, regions implicated in the social cognition network with differing lateralization and localization of activations in patients with MTLE compared to HC. There was apparently greater consistency in activation patterns between HC and patients with left MTLE and hypoactivation, and reduced functional connectivity was generally more pronounced in patients with right MTLE, which further correlated with poorer behavioral performance on social cognition tasks. Specifically, right medial temporal lobe damage was associated with impaired recognition of fear as well as hypoactivation of the social cognition network, and localized amygdala lesions altered the functional pattern of activation in distal regions of the entire social cognition network.

These findings are in agreement with the suggestion that the right medial temporal lobe is preferentially involved in the processing of fear and that related lesions disrupt the overall neural network involved in social cognition. In addition, the left amygdala appears to have a limited capacity to compensate in case of right amygdala damage. Widespread functional disruptions in MTLE are also in line with the new understanding of epilepsy as a network disease.

### Limitations and Recommendations for Future Studies

Assimilating findings from functional neuroimaging studies to identify commonalities is a challenging exercise regardless of the functional domain under study. The reasons for this are multifactorial. fMRI results are highly specific to the paradigms and protocols employed and sensitive to scanner resolution, and the scanning environment and ultimate data analysis platforms and techniques are applied. Results are also dependent on a patient's cognitive functioning of level of participation in the fMRI task during scanning, which is troublesome to control. Nevertheless, reviewing neuroimaging findings makes an important contribution to detecting trends and outstanding neural correlates of affected functional domains in neurological diseases and guiding further research. As such, insights into the mechanisms of social cognitive deficits in people with epilepsy will assist in the management and treatment of these patients in an effort to improve overall QOL.

It will be important for future studies to elaborate on the salient findings presented in this review, particularly interrogating the mechanisms of disturbed social cognition in patients with right MTLE, as well as exploring potential deficits in patients with right frontal and occipital lobe epilepsy, which involve other important structures in social cognition. Future studies could also explore the relationship between poor social cognition in epilepsy and other aspects of cognitive impairments, comorbidities, and access to social support. Resting state functional connectivity is a useful technique to employ in exploring disruptions in the social cognition network across a variety of patient populations, to control for some of the aforementioned confounds related to fMRI. Resting state functional connectivity has been recommended in the study of mechanisms of social cognition in healthy and diseased populations as it shifts the focus from context-dependent aberration to independent aberrations in the functional network architecture. Analyses of RS data would also afford a fairer comparison between studies and findings may be considered in concert with structural connectivity and molecular imaging results (46). Once the neuroimaging literature of social cognition in epilepsy reaches such maturity, meta-analyses using techniques like activation likelihood estimations [ALE; (47)] would lead to valuable insights.

## Author Contributions

All authors listed have made a substantial, direct and intellectual contribution to the work, and approved it for publication.

### Conflict of Interest Statement

The authors declare that the research was conducted in the absence of any commercial or financial relationships that could be construed as a potential conflict of interest.
